# Special Series Guest Editorial: Artificial Intelligence and Machine Learning in Biomedical Optics

**DOI:** 10.1117/1.JBO.26.5.052901

**Published:** 2021-05-10

**Authors:** Behrouz Shabestri, Mark A. Anastasio, Baowei Fei, Frédéric Leblond

**Affiliations:** a National Institute of Biomedical Imaging and Bioengineering, Maryland, United States; b University of Illinois at Urbana–Champaign, Illinois, United States; c University of Texas at Dallas, Texas, United States; d UT Southwestern Medical Center, Texas United States; e Department of Engineering Physics, Polytechnique Montréal, Montreal, Quebec, Canada; f Centre de recherche du Centre hospitalier de l’Université de Montréal, Montreal, Quebec, Canada

## Abstract

Guest editors Behrouz Shabestri, Mark Anastasio, Baowei Fei, and Frédéric Leblond provide an overview of the JBO Special Series on Artificial Intelligence Machine Learning in Biomedical Optics.

Artificial Intelligence (AI) methods, including machine learning (ML) and deep learning (DL), are quickly evolving, and impacting a very wide range of scientific endeavors. Biomedical optics is no exception and AI methods are currently transforming our discipline on an almost daily basis. From changing data acquisition^1^^,^^2^ and image reconstruction methods,^3^ to segmentation and interpretation of optical images,^4^ AI methods are providing improved solutions to established problems and enabling new problems to be addressed.

Structured light can be combined with AI methods to probe and interpret the interaction of light with biological tissues. For example, the coupling of AI methods with hyperspectral and multispectral systems can enable the detection of specific molecular signatures in tissue, cells, and biofluids.^5^^,^^6^ Supervised ML/DL methods are well-suited for this purpose, since they can implicitly learn high-dimensional image statistics and complicated mappings that describe optimal decision strategies for a variety of inferences of relevance to basic science and clinical applications.

Enhancing advanced optical methods with AI will enable the clinical translation of new optical sensing and imaging technologies. Label free optical imaging, such as stimulated Raman histology, hyperspectral imaging, and convolutional neural networks (CNNs), has been successfully employed for intraoperative automated brain tumor diagnosis with near real-time detection.^7^^,^^8^ Integrating ML/DL methods with optical methods such as coherent anti-Stokes Raman scattering imaging, optical colonoscopy and fluorescence lifetime imaging has shown to be effective in the differential diagnosis of lung cancer,^9^ colorectal cancer,^10^ and cervical neoplasia,^11^ respectively. Another AI-enabled game-changer will be the use of DL methods for computational staining of label-free optical images, resulting in all-digital histopathology.^12^^–^^14^

In clinical decision making, where accuracy and timing can be critical, spatial frequency domain imaging coupled with ML has been employed for predicting the severity of burn injuries.^15^ The combination of multi-photon imaging with ML/DL has further enabled improved lymphedema diagnosis,^16^ skin cancer screening^17^ and atopic dermatitis.^18^ ML combined with emerging feature engineering approaches has become the mainstay in tissue, cells, and biofluids interrogation in spectroscopic methods. Examples of such applications range from neurosurgical guidance using spontaneous Raman spectroscopy for cancer detection^19^ to detection of aggressive variants of prostate cancer in pathology using Raman micro-spectroscopy.^20^

Merging optical coherence tomography (OCT) imaging with AI provides a unique opportunity to analyze this plethora of information and assist in making clinical decisions in the field of ophthalmology with applications in retinal imaging,^21^ glaucoma^22^ and age-related macular degeneration.^23^

Most recently, AI methods are proving to be invaluable for a variety of tasks related to the detection and management of COVID-19.^24^^–^^26^ Combining AI with optical breathalyzers may yield a rapid and accurate test for COVID-19, which is currently lacking and greatly needed.

This JBO special series brings together late breaking research that describe the use of artificial intelligence in biophotonic applications, with an emphasis on ML and DL approaches. The series highlights the important role that ML and DL methods are playing in accelerating the development of innovative biophotonic technologies. This series is timely, for it comes as a growing number of the biomedical optics scientific community are starting to tackle the multiple challenges associated with the responsible adoption of AI methods. Issues such as robustness, reliability, and interpretability remain largely unaddressed but are critical for safe and effective deployment of AI-enabled biophotonic imaging and sensing systems. We hope you enjoy this special series, which includes the following twelve articles:

C. Canavesi, A. Cogliati, and H. B. Hindman, “Unbiased corneal tissue analysis using Gabor-domain optical coherence microscopy and machine learning for automatic segmentation of corneal endothelial cells,” doi 10.1117/1.JBO.25.9.092902
A. Hauptmann and B. T. Cox, “Deep learning in photoacoustic tomography: current approaches and future directions,” doi 10.1117/1.JBO.25.11.112903
B. O. L. Mellors et al., “Applications of compressive sensing in spatial frequency domain imaging,” doi 10.1117/1.JBO.25.11.112904
I. Fredriksson, M. Larsson, and T. Strömberg, “Machine learning for direct oxygen saturation and hemoglobin concentration assessment using diffuse reflectance spectroscopy,” doi 10.1117/1.JBO.25.11.112905
D. S. Gareau et al., “Deep learning-level melanoma detection by interpretable machine learning and imaging biomarker cues,” doi 10.1117/1.JBO.25.11.112906
M. Chen and N. Durr, “Rapid tissue oxygenation mapping from snapshot structured-light images with adversarial deep learning,” doi 10.1117/1.JBO.25.11.112907
B. Lyu et al., “Domain adaptation for robust workload level alignment between sessions and subjects using fNIRS,” doi 10.1117/1.JBO.26.2.022908
S. Guo et al., “FLIM data analysis based on Laguerre polynomial decomposition and machine-learning,” doi 10.1117/1.JBO.26.2.022909
M. S. Durkee et al., “Quantifying the effects of biopsy fixation and staining panel design on automatic instance segmentation of immune cells in human lupus nephritis,” doi 10.1117/1.JBO.26.2.022910
F. Daoust et al., “Handheld macroscopic Raman spectroscopy imaging instrument for machine learning based molecular tissue margins characterization,” doi 10.1117/1.JBO.26.2.022911
M. H. Nguyen et al., “Machine learning to extract physiological parameters from multispectral diffuse reflectance spectroscopy,” doi 10.1117/1.JBO.26.5.052912
B. X. Guan et al., “Human embryonic stem cell classification: random network with autoencoded feature extractor,” doi 10.1117/1.JBO.26.5.052913.
